# Improvement of chicken plasma protein hydrolysate angiotensin I‐converting enzyme inhibitory activity by optimizing plastein reaction

**DOI:** 10.1002/fsn3.1572

**Published:** 2020-05-18

**Authors:** Dandan Gao, Penghui Guo, Xin Cao, Lili Ge, Hongxin Ma, Hao Cheng, Yiqiang Ke, Shien Chen, Gongtao Ding, Ruofei Feng, Zilin Qiao, Jialin Bai, Nurul I. Nordin, Zhongren Ma

**Affiliations:** ^1^ China‐Malaysia National Joint Laboratory Biomedical Research Center Northwest Minzu University Lanzhou P. R. China; ^2^ College of Life Sciences and Engineering Northwest Minzu University Lanzhou P. R. China; ^3^ Industrial Biotechnology Research Centre SIRIM Berhad Selangor Malaysia

**Keywords:** ACE inhibitory activity, chicken plasma protein, hydrolysate, plastein reaction, response surface methodology

## Abstract

Chicken plasma protein hydrolysate (CPPH) was prepared by trypsin with angiotensin I‐converting enzyme (ACE) inhibitory activity of 53.5% ± 0.14% and the degree of hydrolysis (DH) of 16.22% ± 0.21% at 1 mg·ml^−1^; then, five proteases, including pepsin, trypsin, papain, alcalase, and neutrase, were employed to improve ACE inhibitory ability by catalyzing plastein reaction. The results indicated that trypsin‐catalyzed plastein reaction showed the highest ACE inhibitory activity. The exogenous amino acids of leucine, histidine, tyrosine, valine, and cysteine were selected to modify the CPPH. The leucine‐modified plastein reaction released the highest ACE inhibitory activity. The effects of four reaction parameters on plastein reaction were studied, and the optimal conditions with the purpose of obtaining the most powerful ACE inhibitory peptides from modified products were obtained by response surface methodology (RSM). The maximum ACE inhibition rate of the modified hydrolysate reached 82.07% ± 0.03% prepared at concentration of hydrolysates of 30%, reaction time of 4.9 hr, pH value of 8.0, temperature of 40°C, and E/S ratio of 5,681.62 U·g^−1^. The results indicated that trypsin‐catalyzed plastein reaction increased ACE inhibitory activity of chicken plasma protein hydrolysates by 28.57%.

## Graphical abstract

Chicken plasma protein was hydrolyzed by trypsin to obtain CPPH with ACE inhibitory activity, and the hydrolysates were modified by a trypsin‐catalyzed plastein reaction to improve its ACE inhibitory activity. The reaction conditions of trypsin‐catalyzed plastein reaction of CPPH were optimized by single‐factor experiments and response surface methodology, obtaining optimal condition for improving ACE inhibitory activity. The result showed that the trypsin‐catalyzed plastein reaction of CPPH could enhance ACE inhibitory activity of modified hydrolysates, indicating that chicken plasma protein has the potential in the development of new functional foods.

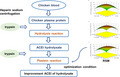

## 1 INTRODUCTION

Hypertension is a common disease among humans and often leads to cardiovascular disease (Fahmi et al., 2004). ACE is a key enzyme in the renin–angiotensin system, acting an important part in controlling the blood pressure (Sieber et al., 2010). The synthetic ACE inhibitors, such as captopril, enalapril, and lisinopril, have been widely used in clinical to treat hypertension disease (Barbosa‐Filho et al., 2006). However, the synthetic ACE inhibitors have some side effects, including skin rashes, coughing, and taste disturbances (FitzGerald, Murray, & Walsh, 2004). Food‐derived ACE inhibitory peptides are safer and milder than synthetic ones. Since the first report (Oshima & Nagasawa, 1979), many food‐derived ACE inhibitory peptides have been separated from various food proteins, such as soya protein (Tomatsu, Shimakage, Shinbo, Yamada, & Takahashi, 2013), rice protein (He, Xuan, Ruan, Chen, & Xu, 2005), cottonseed protein (Gao, Chang, Li, & Cao, 2010), egg proteins (Ren et al., 2008a), fish proteins (Raghavan & Kristinsson, 2009), sour milk (Pan & Guo, 2010), and hemoglobin proteins (Mito et al., 1996). Food‐derived ACE inhibitory peptides have a high development prospects. However, food‐derived ACE inhibitory peptides have lower activity and stability compared with synthetic ACE inhibitors.

The plastein reaction is a process in which a protease catalyzes the formation of a precipitate, thixotropic colloid, or thixotropic viscous gel with a high concentration of protein hydrolysate, which can be simply regarded as a reverse reaction of a proteolytic reaction (Yamashita, Arai, Tsai, & Fujimaki, 1971). The plastein reaction has been applied in debittering treatment for protein hydrolysates (Fujimaki, Yamashita, Arai, Kato, & Gonda, 1970), amino acid fortification (Gong, Mohan, Gibson, & Udenigwe, 2015), and property modification for protein ingredients. Some researchers also found that plastein reaction could enhance bioactivity of protein hydrolysates. A previous study showed that sea cucumber hydrolysates modified by plastein reaction of the exogenous proline increased the ACE inhibitory activity of natural peptides (Li, Liu, et al., 2018). Gao and Zhao (2012) reported that alcalase‐catalyzed plastein reaction could be applied as a potential approach to enhance the ACE inhibitory activity of soybean protein hydrolysates in vitro. Wei, Li, and Zhao (2013) demonstrated that neutrase‐catalyzed plastein reaction enhanced the ACE inhibitory activity of casein hydrolysates.

Chicken blood is a by‐product of slaughterhouses and contains several proteins. It can be used in both feed and food industries owing to good nutritional value and excellent functional properties (Rawdkuen, Benjakul, Visessanguan, & Lanier, 2004). However, only a small proportion of chicken blood has currently been recycled as feed, food, or fertilizer (Lasekan, Bakar, & Hashim, 2013). In recent years, chicken blood has gradually garnered attention for use as a raw material for the preparation of antioxidant peptides (Zheng, Si, Ahmad, Li, & Zhang, 2018) and protease inhibitors (Lasekan et al., 2013).

In the present study, chicken plasma protein was hydrolyzed by trypsin, showing the potential for preparing ACE inhibitory peptides. This study aims to enhance the ACE inhibitory activity of chicken plasma protein hydrolysate (CPPH) by plastein reaction. Some factors affecting plastein reaction, including different protease, different exogenous amino acids, ratio of enzyme to substrate (E/S), reaction temperature, pH value, and time, were evaluated by single‐factor trials and Box–Behnken Design. The ACE inhibitory activities of modified products were analyzed and compared with that of the original hydrolysate, revealing the impacts of plastein reaction on ACE inhibitory activity of the modified product of CPPH.

## 2 MATERIALS AND METHODS

### 2.1 Materials

Chicken blood was obtained from a slaughterhouse in Lanzhou, China. During collection, 4.0% (w/v) heparin sodium was added to prevent coagulation. Pepsin, trypsin, papain, hippuryl‐histydyl‐leucine (HHL), ACE were purchased from Sigma‐Aldrich Chemical Co. neutrase, alcalase, cysteine, tyrosine, leucine, histidine, valine, and glycine were purchased from Zhongqin Chemical Reagent Co., Ltd. Ninhydrin was purchased from Soulebao Biological Reagent Co., Ltd. Other reagents used were chemicals of analytical grade.

### 2.2 Preparation of chicken plasma protein

Chicken plasma protein was separated by centrifugation at 8,000 rpm and 4°C for 5 min (J‐301, Beckman), and the supernatant was collected and freeze‐dried before further use.

### 2.3 Preparation of chicken plasma protein hydrolysate

Chicken plasma protein (5 g) was dissolved in 100 ml deionized water to give an original protein concentration of 5% (w/v). The pH of solution was adjusted to 7.5 by 1 M NaOH. Hydrolysis reaction was started by adding trypsin to chicken plasma protein solution to give an E/S ratio of 6,000 U·g^−1^ protein followed by incubation for 5 hr at 37°C. After the hydrolysis reaction finished, the solutions were heated at 95°C for 15 min to inactivate trypsin, cooled to room temperature, and centrifuged at 10,000 rpm for 20 min. The obtained supernatants were analyzed to determine the corresponding ACE inhibitory activity in vitro, then were lyophilized and stored at −20°C, and used as substrate of the plastein reaction (Jang & Lee, 2005).

### 2.4 Modification of chicken plasma protein hydrolysate by plastein reaction

The plastein reaction on CPPH was carried out with five different proteases (pepsin, trypsin, papain, alcalase, and neutrase) and exogenous amino acids (leucine, histidine, tyrosine, valine, and cysteine), respectively. The reaction parameters of different enzyme, recommended by their product manual, are shown in Table 1. In the modification process, the substrate concentration was fixed at 30%, and proteases and exogenous amino acids were added in the hydrolysate solution, respectively. Then, the samples were prepared at different temperatures, pH levels, E/S ratio, and time periods. Reactions were stopped by heating the solution in boiling water for 15 min, and the samples were cooled to room temperature and centrifuged at 10,000 rpm for 5 min. Finally, the supernatant was analyzed to determine the ACE inhibitory activity in vitro and the decreased amount of free amino groups, lyophilized before further use.

**Table 1:** Plastein reaction condition parameters of different enzymes

Protease	Concentration of hydrolysates	E/S ratio	Temperature	pH value	Time
Pepsin	30%	6,000 U·g^−1^	37°C	1.8	4 hr
Neutrase	30%	6,000 U·g^−1^	45°C	6.5	4 hr
Alcalase	30%	6,000 U·g^−1^	50°C	8.0	4 hr
Trypsin	30%	6,000 U·g^−1^	40°C	7.5	4 hr
Papain	30%	6,000 U·g^−1^	50°C	5.7	4 hr

### 2.5 Experimental design

In order to improve the ACE inhibitory activity of protein hydrolysate, trypsin‐catalyzed plastein reaction with leucine was adopted in the modification of chicken plasma protein peptides because its plastein reaction product had the highest ACE inhibitory activity. Single‐factor experiments and a Box–Behnken design were used to determine the optimal conditions of trypsin‐catalyzed plastein reaction with leucine. In single‐factor experiments, different reaction conditions such as reaction temperatures (35, 40, 45, 50, 55°C), pH values (7.0, 7.5, 8.0, 8.5, 9.0), E/S ratio (4,000, 5,000, 6,000, 7,000, 8,000 U·g^−1^), and reaction times (4.0, 4.5, 5.0, 5.5, 6.0 hr) were carried out for optimizing the plastein reaction. Based on the single‐factor experiments, a Box–Behnken design was used to determine the optimal conditions of trypsin‐catalyzed plastein reaction with leucine, with the ACE inhibitory activity of modification product as the response value, and the reaction time as factor A, pH value as factor B, and E/S ratio as factor C. The experimental design levels for the response surface are exhibited in Table 2. In addition, multiple regression analysis through the quadratic method was performed, and the data were fitted into an empirical second‐order polynomial equation (Mune, Minka, & Mbome, 2008). The equation is given as:

$$Y=b_{0}+b_{1}A+b_{2}B+b_{3}C+b_{11}A^{2}+b_{22}B^{2}+b_{33}C^{2}+b_{12}AB+b_{13}AC+b_{23}BC$$

where *Y* is the dependent variables (ACE inhibitory activity), *b*_0_ is an offset term, *b*_1_, *b*_2_, and *b*_3_ are the linear regression coefficients, *b*_11_, *b*_22_, and *b*_33_ are quadratic regression coefficients and *b*_12_, *b*_13_, and *b*_23_ are interaction regression coefficients, and *A*, *B*, and *C* are levels of the independent variables.

**Table 2:** Variables and experimental design levels for response surface

Independent variables	Coded factor levels		
	−1	0	1
A: Time (h)	4.5	5	5.5
B: pH value	7.5	8	8.5
C: E/S ratio(U·g^−1^)	5,000	6,000	7,000

### 2.6 Determination of amount of free amino groups

The amount of free amino groups of the modified product was measured by ninhydrin colorimetry assay with minor modifications (Doi, Shibata, & Matoba, 1981). In our study, glycine was used as a standard. A series of diluted glycine solutions in the range of 0–20 μg⋅ml^−1^ were prepared by further dilution and used to determine the standard curve. The ninhydrin solution was prepared as following: weighed 1.5 g ninhydrin into a beaker and mixed with 15 ml of *n*‐propanol, 30 ml of n‐butanol, 60 ml of ethylene glycol, and 9 ml of acetate buffer (pH = 4.54). The further test was carried out by adding sample solution of 3 ml to 1 ml ninhydrin solution and 5 ml of 40% ethanol solution. The absorbance was measured at 570 nm with an UV‐2600 spectrophotometer (Shimadzu Ltd). The amount of free amino groups of the sample was calculated according to the following formula :

$$Y=\frac{C\times N}{m\times 75.07}$$

where *Y* is amount of free amino groups of the sample, mmol·g^−1^; C is amount of free amino groups of standard curve, µg; N is sample dilution factor; *m* is sample weight, g; 75.07 is the molar mass of glycine, g·mol^−1^.

### 2.7 Determination of ACE inhibitory activity

The assay for ACE inhibition was performed as the method of Cushman and Cheung (Cushman & Cheung, 1971) with some modifications. The HHL was dissolved in 0.1 M borate buffer containing 0.3 M NaCl (pH8.3) to prepare a concentration of 5 mM. Then, 150 μL of 5 U⋅ml^−1^ ACE was added to the mixture and incubated at 37°C for 60 min. After incubation, the reaction mixture was stopped by adding 250 μl of 1 M HCl and then added 1.5 ml of ethyl acetate, after strong oscillation for 30 s by a HY‐1 vortex oscillator (Leici Instrumentation Company), centrifugated at 10,000 rpm for 10 min. Then, 1 ml of ethyl acetate layer was taken off and completely dried at 120°C for 30 min. The residue was dissolved in 3.0 ml of distilled water and cooled to room temperature. The absorbance was determined at 228 nm in an UV‐2600 spectrophotometer (Shimadzu Ltd). Each sample was essayed in triplicate. The ACE inhibitory activity rate was calculated as follows:

$$\text{ACE inhibitory activity}\left(\%\right)=\frac{A-B}{A-C}\times 100\%$$

where A is absorbance of the control, B is absorbance of sample, and C is absorbance of blank.

### 2.8 Determination of DH

The DH of CPPH was determined using the ninhydrin colorimetric method with minor modifications (Pearce, Karahalios, & Friedman, 1988). In this study, glycine was used as the standard instead of leucine. After hydrolysis, 1 ml fresh ninhydrin reagent (ninhydrin reagent was made by dissolving 1.5 g ninhydrin in 10 ml acetate buffer, then adding 60 ml ethylene glycol, 15 ml n‐propanol and 15 ml n‐butyl alcohol.) was mixed with 3 ml CPPH solution followed by heating for 20 min, the mixture was cooled in an ice bath, and 1 ml ethanol solution (40%, v/v) was then supplemented. The absorbance at 570 nm was measured in an UV‐2600 spectrophotometer. The DH value was calculated as follows:

$$\text{DH}\;\left(\%\right)\;=\frac{A_{N2}-A_{N1}}{A_{N0}}\times 100.$$

where *A*_N2_ is the concentration of free amino nitrogen of hydrolysate (µmol·g^−1^); *A*_N1_ is the concentration of free amino nitrogen of protein (µmol·g^−1^); *A*_N0_ is the concentration of total amino nitrogen of protein (µmol·g^−1^).

### 2.9 Statistical analysis

Design Expert software (Version 8.0.6, Stat‐Ease, Inc.) was used for the experiment design and ANOVA. All experiments or analyses were carried out in triplicate, and the data were reported as means and standard deviations. Differences between the mean values of multiple groups were analyzed by one‐way analysis of variance and *F*‐test to the significance of the statistical model.

## 3 RESULTS AND DISCUSSION

### 3.1 Effects of proteases on plastein reaction of CPPH

**Figure 1:**
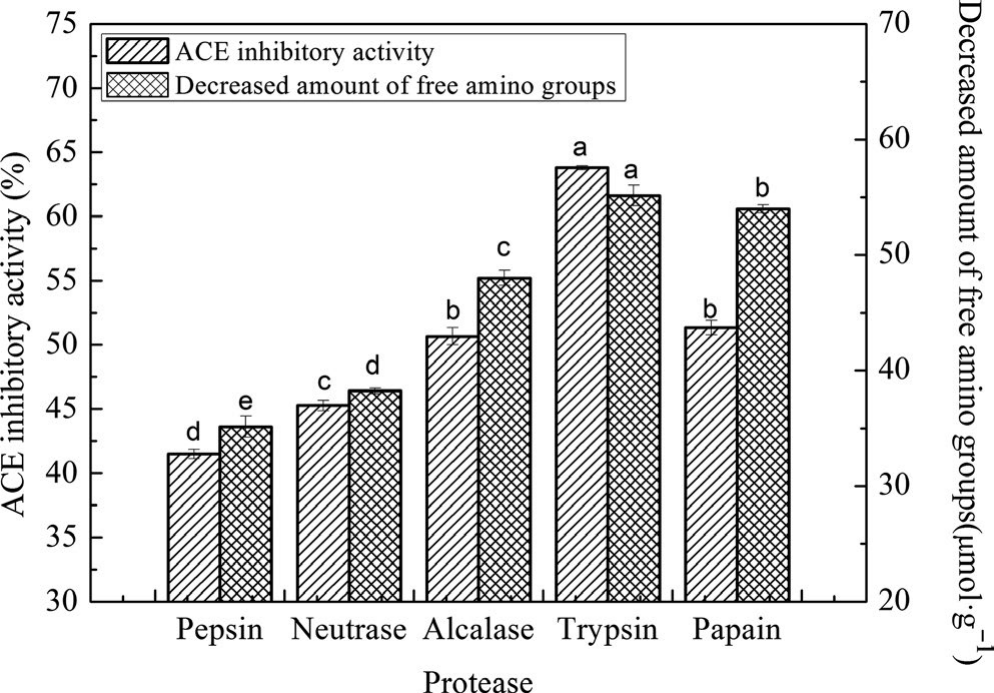
Effects of different proteases on plastein reaction

The chicken plasma protein hydrolysate prepared in the present study had a DH of 16.22% ± 0.21% and ACE inhibitory activity of 53.5% ± 0.14%. When the hydrolysate was treated by plastein reaction catalyzed with different proteases at recommended conditions by each operational instruction, substrate concentration 30%, the results (Figure 1) showed that the plastein reaction catalyzed with trypsin and papain could significantly increase ACE inhibitory activities and decrease amount of free amino groups of treated hydrolysates. The decrease of free amino groups of the modified products using different proteases were as follows: trypsin (55.14 ± 0.87 µmol·g^−1^), pepsin (35.12 ± 0.91 µmol·g^−1^), neutrase (38.23 ± 0.21 µmol·g^−1^), alcalase (48.01 ± 0.64 µmol·g^−1^), and papain (53.98 ± 0.36 µmol·g^−1^), respectively, implying that peptide condensation occurred during plastein reaction. In addition, the product produced by trypsin‐catalyzed plastein reaction exhibited strongest ACE inhibitory activities (63.8% ± 0.16%), and the others in turn was papain (51.33% ± 0.56%), alcalase (50.67% ± 0.66%), neutrase (45.27% ± 0.41%), and pepsin (41.5% ± 0.37%). The results indicated that trypsin was the best protease using to modify chicken plasma protein hydrolysate in plastein reaction. Plastein reaction is multiple, including continuous hydrolysis and aggregation of peptides which might happen simultaneously. If the most susceptible peptide bonds had already been cleaved by trypsin during hydrolysis of CPPH, hydrolysates would have more opportunity to form protein‐like plasteins when being catalyzed by the same enzyme. This maybe the reason why the modified product produced by trypsin‐catalyzed plastein reaction exhibited strongest ACE inhibitory ability. Qian et al. (2018) found that pepsin‐catalyzed plastein reaction could enhance bile acid‐binding capacity of soybean protein hydrolysates and whey protein hydrolysates which were also hydrolyzed by pepsin. Hui and Zhao (2009) reported that casein hydrolysate which prepared by alcalase and was modified by the alcalase‐catalyzed plastein reaction could improve its ACE inhibitory activity in vitro. Wei et al. (Wei et al., 2013) found that casein hydrolysates were prepared by alcalase and showed ACE inhibition in vitro with an IC_50_ value of 45.2 µg·ml^−1^. Then, the hydrolysates were modified by plastein reaction catalyzed by neutrase to investigate the impact of the coupled neutrase‐catalyzed plastein reaction on the ACE inhibition of the casein hydrolysates. The results revealed that the coupled neutrase‐catalyzed plastein reaction improved the ACE inhibition of modified hydrolysates with IC_50_ values 15.6 µg·ml^−1^. These studies indicated the protease of plastein reaction could be chosen as the same as the hydrolysis reaction and could also be different with the hydrolysis reaction. In our study, trypsin was selected for further study on plastein reaction modification of CPPH.

### 3.2 Effects of exogenous amino acids on plastein reaction of CPPH

**Figure 2:**
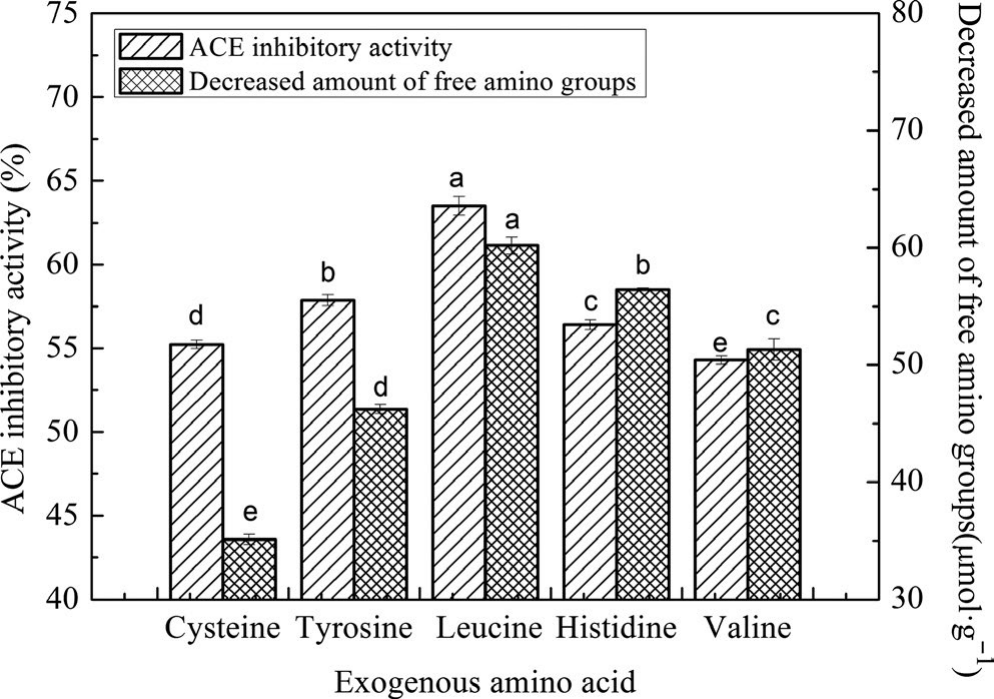
Effects of different exogenous amino acids on plastein reaction

The exogenous amino acids of leucine, histidine, tyrosine, valine, and cysteine were used to modify the ACE inhibitory peptides from chicken plasma protein, respectively. As shown in Figure 2, the product modified by trypsin‐catalyzed plastein reaction with leucine exhibited the maximum ACE inhibitory activity of 63.5% ± 0.57% and showed the decreased amount of free amino groups of 60.19 ± 0.72 µmol·g^−1^. The ACE inhibition rates of modified product by other exogenous amino acids in turn were tyrosine (57.87% ± 0.33%), histidine (56.4% ± 0.29%), cysteine (55.23% ± 0.26%), and valine (54.3% ± 0.24%). The binding of ACE inhibitory peptides to ACE was strongly influenced by the C‐terminal sequence of the peptides. Wu et al. (Wu, Aluko, & Nakai, 2006) found that leucine which is a hydrophobic amino acid at the C‐terminus of the peptides will be exhibited more ACE inhibitory activity, according to the quantitative structure–activity relationships for the ACE inhibitory peptide models. Structure–activity relationship analysis also implied that amino acids Ile, Leu, Ala, and Val were preferred at the N‐terminus, whereas Phe, Trp, and Tyr were preferred at the C‐terminus had a positive effect on potency of ACE inhibitory peptides (Toopcham, Mes, Wichers, Roytrakul, & Yongsawatdigul, 2017). This could explain high ACE inhibitory ability of product modified by trypsin‐catalyzed plastein reaction with leucine may consist of leucine at the N‐terminus and the C‐terminal.

### 3.3 Effects of reaction E/S ratio, temperature, pH value, and reaction time on plastein reaction of CPPH

**Figure 3:**
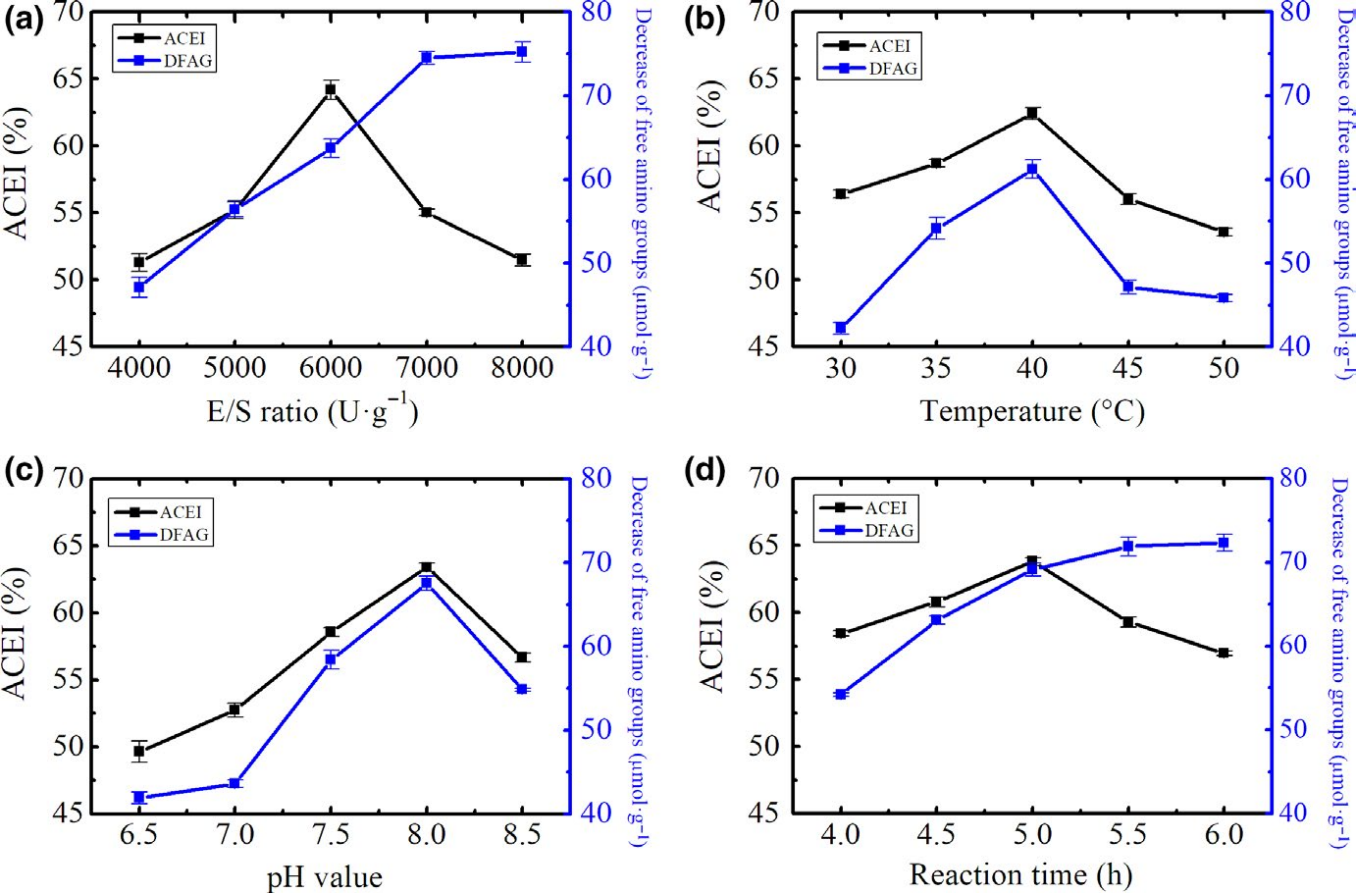
Effects of E/S ratio (a), temperature (b), pH (c), and time (d) on plastein reaction

Plastein reaction undertakes usually at higher substrate concentration ranging from 20% to 50% by weight (Sukan & Andrews, 1982). In the present  study,  we used trypsin‐catalyzed plastein reaction with leucine to modify the substrate CPPH，with a concentration of 30% (w/w) in water. Figure 3a reveals the effects of E/S ratio on ACE inhibitory ability of the modified products. The free amino groups was decreased when the E/S ratio varied from 4,000 to 8,000 U·g^−1^ and reached 75.2 ± 1.2 µmol·g^−1^ at E/S ratio of 8,000 U·g^−1^. Differently, ACE inhibitory ability reached a maximum of 63.1% ± 0.08% when E/S ratio was 6,000 U·g^−1^, and the further increase of E/S caused the decrease of the value of ACE inhibitory rate of modified product. In our study, the decrease of free amino groups which indeed was an indicator of condensation was used to reflect the extent of plastein reaction of CPPH. As the E/S ratio increased, the degree of plastein reaction was maximized; however, the ACE inhibitory rate of the modified product simultaneously decreased, possible because the E/S ratio was too high, and too much protease easily catalyzed the re‐hydrolysis reaction of the product; the reduction of the active substance leads to a decrease in the ACE inhibitory rate. Therefore, the E/S ratio of 6000U·g^−1^ was chosen as central point in Box–Behnken design of the response surface.

The effects of plastein reaction temperature on ACE inhibitory ability and decrease of free amino groups were determined with the reaction time 4 hr, pH 7.0, and E/S ratio 6000U·g^−1^. Under this condition, five modified products with different reaction extents were prepared at 30–50°C. The ACE inhibitory ability and decrease of free amino groups of these treated hydrolysates of CPPH are shown in Figure 3b. The ACE inhibitory activities and decrease of free amino groups of the modified products showed an increasing trend as temperature incremented from 30 to 40°C; after then, both the ACE inhibitory activity and decrease of free amino groups were decreased with temperature from 40 to 50°C. A reaction temperature of 40°C conferred the highest ACE inhibitory activity (62.4% ± 0.43%) and the maximum decrease of free amino groups (61.22 ± 1.12 µmol·g^−1^) on the treated hydrolysate. Differently, Wu, Xu, Sun, Yu, and Zhou (2014) reported that marine *I. galbana* protein displayed high ACE inhibitory activity after hydrolysis by trypsin at 55.64°C. An active protease is important to catalyze plastein reaction. The range of reaction temperature was restricted by the optimal catalytic temperature of the enzyme used. Lower temperature is beneficial as plastein reaction is an exothermic reaction (Fujimaki, Kato, Arai, & Yamashita, 1971), while higher temperature could slow down even stop the reaction immediately, although the initial rate of the plastein reaction was rapid.  Therefore, higher reaction temperature might not be a suitable selection. Considering heat stability of trypsin and reaction rate of the plastein reaction, temperature was fixed at 40°C in later work.

The effects of pH from 7.0 to 9.0 on ACE inhibitory ability and free amino groups were investigated. The substrate concentration, E/S ratio, temperature, and time of trypsin‐catalyzed plastein reaction were set at 30%, 40°C, 6,000 U·g^−1^, and 4.0 hr, respectively. As the reaction progressed from pH of 7.0 to 9.0, the ACE inhibitory rate and free amino groups firstly increased and then decreased; for pH 8.0, the ACE inhibitory rate and free amino groups both could reach the maximum at 63.4% ± 0.33% and 67.52 ± 0.82 µmol·g^−1^, respectively (Figure 3c). This was possibly because the ability of trypsin could not be activated in surroundings with alkali. The pH of the reaction medium was also an important factor influencing plastein formation. Ferreira et al. (2007) found that whey protein hydrolysates obtained from tryptic hydrolysis showed ACE inhibitory activity with IC_50_ value of 42.6 mM at pH 8.0. The present result shared similarity to this study. Xue et al (Xue et al., 2018) reported that an ACE inhibitory peptide was isolated from the trypsin hydrolysate of bovine casein at pH 7.5. Due to the acidic or alkaline environment, proteases and substrate proteins were degraded to a certain degree, causing the proteases to lose some of the catalysis function, and reduced the ACE inhibitory ability. Hence, the central point was sited at pH of 8.0 with 0.5 for step changes in Box–Behnken design.

The impacts of reaction time on the plastein reaction are shown in Figure 3d. The ACE inhibitory activity of modified products increased with the time from 4.0 to 5.0 hr; after 5.0 hr, the ACE inhibitory activity reached the maximum value of 63.8%. The reaction time continued to prolong, and the ACE inhibitory rate decreased. The decreased amount of free amino groups of modified products increased linearly (*p* < .05) from 4.0 to 5.0 hr and then reached a plateau with decreased amount of free amino groups of 69.12–72.33 µmol·g^−1^ during 5.0–6.0 hr. The decreased amount of free amino groups of the modified hydrolysate in the present study increased rapidly at initial stage of reaction and then showed a decreased trend, which was similar to another study (Xu, Kong, & Zhao, 2014). In present study, we found that when CPPH was catalyzed for 5 hr, the modified hydrolysate showed the most ACE inhibitory activity of 63.8% ± 0.28%, but longer hydrolysis time led the modified hydrolysate lost their ability to inhibit ACE. The further treatment would increase molecular size of ACE inhibitory peptides and thus destroyed its ACE inhibitory activity. Therefore, 5.0 hr was ensured as the optimum plastein reaction time.

## 4 MODEL FITTED AND STATISTICAL ANALYSIS OF RESPONSE SURFACE EXPERIMENTS

RSM had been used in some studies to optimize the hydrolysis conditions of different food proteins, such as the preparation of hydrolysate from cottonseed protein (Gao et al., 2019), black soybean (Li, Xia, Zhang, & Li, 2018), squid protein (Sivaraman et al., 2016), duck blood corpuscle (Zheng et al., 2014), whey protein (Tavares et al., 2011), pumpkin oil cake (Peričin, Radulović‐Popović, Vaštag, Mađarev‐Popović, & Trivić, 2009), and fish protein (Ren et al., 2008b). Xu et al. (2014) optimized some reaction conditions of Neutrase‐catalyzed plastein reaction to modify casein hydrolysate prepared for improving its ACE inhibitory activity in vitro by RSM with a central composite design (CCD). The present study employed the RSM with a Box–Behnken design (BBD) to select suitable reaction time, pH, and the E/S ratio for the trypsin‐catalyzed plastein reaction of the CPPH. The BBD comprises a specific subset of the factorial combinations from the 3^k^ factorial design, and the experimental points are situated on a hypersphere equally distant from the central point. Application of this design is popular in food processes due to its economical design (Xu et al., 2014). The results indicated that the experimental ACE inhibitory rate values fall in the range 75.40%–82.60% (Table 3). The analysis of variance (ANOVA) for the regression model is summarized in Table 4, which was analyzed using Design Expert 8.06 software. The significance of each coefficient was also determined using *F* values and *p* values (Table 4). Values of “Prob > *F*” less than 0.05 indicated model terms were significant. In this case, A, B, C, A^2^, B^2^, C^2^ are significant model terms. Variables with the largest effects were the quadratic terms of time (A^2^), pH (B^2^), E/S ratio (C^2^). Linear terms of time (A) and linear terms of E/S ratio (C) (*p* < .001) were also significant (*p* < .05). Other term coefficients were not significant (*p* > .05).

**Table 3:** Box–Behnken design of the independent variables and experimental values of ACE inhibitory activity (Y)

Run No	Coded levels of variables					ACE inhibitory rate (%)	
	A		B		C	Experimental	Predicted
1		−1		−1	0	78.16 ± 0.64	78.23
2		1		−1	0	76.42 ± 0.45	75.90
3		−1		1	0	76.60 ± 0.67	77.11
4		1		1	0	76.40 ± 0.09	76.32
5		−1		0	−1	79.64 ± 0.52	75.59
6		1		0	−1	76.40 ± 0.86	76.94
7		−1		0	1	76.46 ± 0.34	75.92
8		1		0	1	75.40 ± 0.54	75.45
9		0		−1	−1	78.60 ± 0.75	78.57
10		0		1	−1	79.24 ± 0.71	78.78
11		0		−1	1	76.08 ± 0.54	76.54
12		0		1	1	75.62 ± 0.42	75.64
13		0		0	0	81.46 ± 0.05	81.79
14		0		0	0	82.45 ± 0.48	81.79
15		0		0	0	82.60 ± 0.59	81.79
16		0		0	0	81.06 ± 0.14	81.79
17		0		0	0	81.40 ± 0.64	81.79

**Table 4:** Analysis of variance for ACE inhibitory activity (Y)

Source	Sum of squares	Mean square	*F value*	*p value*
Model	99.32	11.04	22.40	.0002**
A—Time	4.87	4.87	9.88	.0163*
B—pH value	0.24	0.24	0.50	.5035
C—E/S ratio	13.31	13.31	27.02	.0013**
AB	0.59	0.59	1.20	.3090
AC	1.19	1.19	2.41	.1644
BC	0.30	0.30	0.61	.4590
A^2^	29.67	29.67	60.21	.0001**
B^2^	21.21	21.21	43.05	.0003**
C^2^	19.73	19.73	40.04	.0004**
Residual	3.45	0.49		
Lack of fit	1.56	0.52	1.11	.4445
Pure error	1.89	0.47		
Cor total	102.77			
*R* ^2^	0.9664			
Adj *R* ^2^	0.9233			

* represents significant difference (*p < .05*).

** represents extremely significant difference (*p < .01*)

The probability P value was less than 0.05 indicated that the model was significant. Lack of fit analysis was used to measure the adequacy of fit. An *F* value was 1.11 and a P value was 0.44445 showed that the lack of fit was not significant, which indicated that the model was sufficiently accurate. The value of determination coefficient (*R* ^2^) was 0.9664, indicating that 96.64% of the variation could be explained by the fitted model. The adjusted determination coefficient (R_Adj_^2^) is 0.9233, highly enough to advocate the significance of the model. To study the influences of the reaction time (A), pH value (B), and the E/S ratio(C) on the ACE inhibitory ability of the modified products, the experimental data were analyzed with application of multiple regression, and the response variable and test variables were related by a second‐order polynomial equation:

$$Y=81.79-0.78A-0.17B-1.29C+0.38AB+0.55AC-0.27BC-2.65A^{2}-2.24B^{2}-2.16C^{2}$$

where *Y* is the predicted ACE inhibitory activity in real value, *A*, *B*, and *C* are the reaction time, pH value, and the E/S ratio, respectively.

### 4.1 Analysis of the response surface

**Figure 4:**
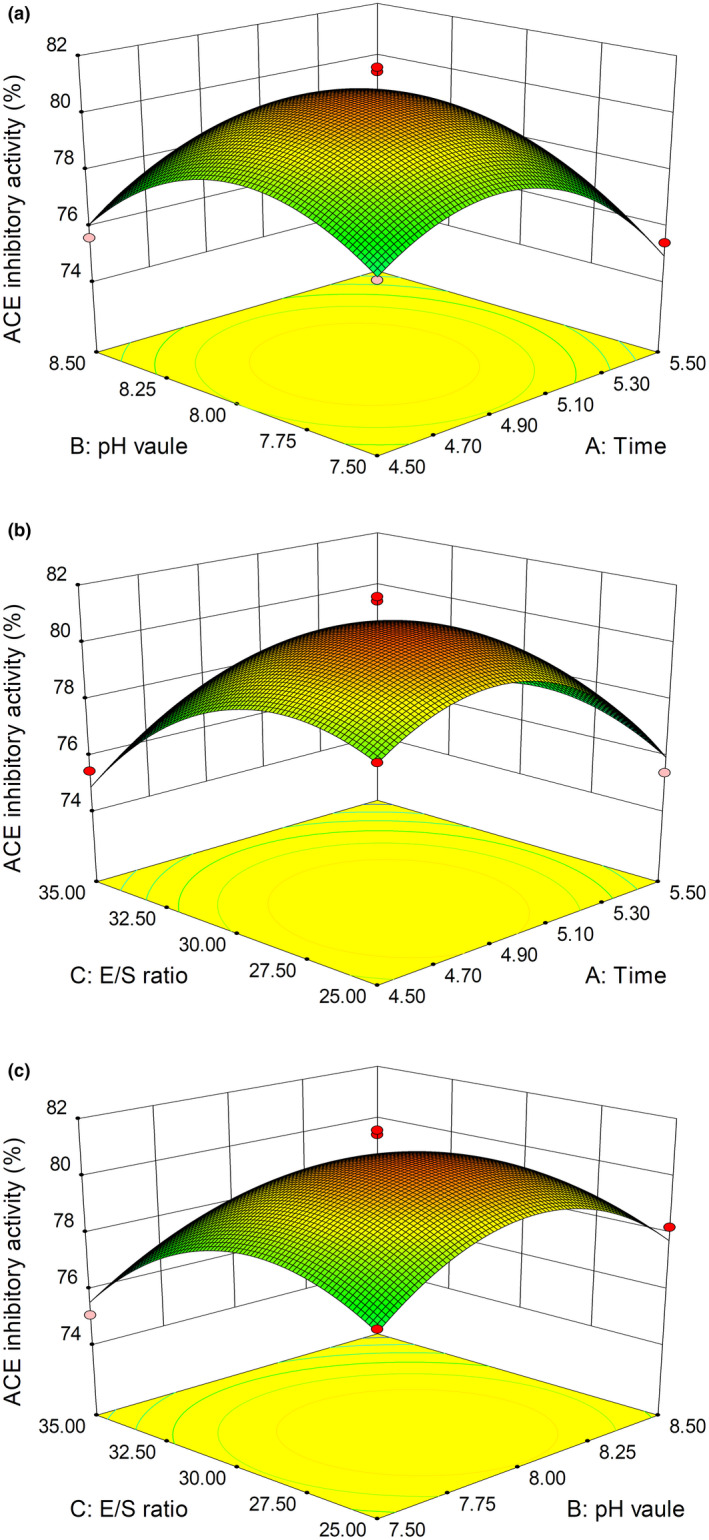
Response surface three‐dimensional plots of the effects of time and pH (a); time and E/S ratio (b); pH value and E/S ratio (c) on the ACE inhibitory activity of modified product

Three‐dimensional response surface plots were used to analyze the effects of independent variables on the activity of plastein reaction (Figure 4). The interaction effects of two factors on the response were investigate while the other one factor was kept at 0 level. An increase in the rate with interactions between pH value and time, E/S ratio and time, and pH and E/S ratio was observed (Figure 4). These increases in the ACE inhibitory rate occurred only up to a maximum point, beyond this point the ACE inhibitory rate decreased.

Figure 4a shows the effects of pH and time on the ACE inhibitory ability of the modified hydrolysate (E/S ratio at its zero level). Quadratic effect for two variables existed, which was similarity to the previous observation of Gao and Zhao (2012). The ACE inhibitory ability was firstly increased and then gradually decreased with pH and time. A larger ACE inhibitory activity of 82.45% occurred when pH was 8 and time was 5 hr.

As shown in Figure 4b, the effects of E/S ratio and reaction time on the ACE inhibitory ability of the modified hydrolysate (pH at its zero level). Quadratic effects of E/S ratio and reaction time on the response were also noticed. Under E/S ratio of 6,000 U·g^−1^ and time of 5 hr, the ACE inhibitory activity reached 82.45%.

In Figure 4c, the ACE inhibitory activity appeared to increase firstly and then decrease with E/S. This might be due to the excessive hydrolysis of the modified product by the protease, reducing the amount of ACE inhibitory active substances in the system, and thus affected the ACE inhibitory ability of the modified product. When pH was 8.0 and E/S was 6,000 U·g^−1^, the ACE inhibitory could reach 82.6%. Cao, Zhang, Wang, and Zhao (2018) reported that the ACE inhibitory peptides from oyster hydrolysate were modified by plastein reaction and the modified product showed maximum ACE inhibitory activity of 82.31%, similarity to our result.

## 5 VALIDATION OF THE PREDICTIVE MODEL

According to the results of the Box–Behnken design, the optimal reaction conditions for each factor with the maximum ACE inhibitory rate (Y) was obtained by the Design Expert software: 4.91 hr, pH 7.98, E/S 5,682.12 U·g^−1^, and the model produced a predicted value for the ACE inhibitory activity of 82.07%. To verify suitability of the model used for prediction of optimum response values, an optimization study was performed. At the optimal condition, the experimental ACE inhibitory activity was 82.05% ± 0.05%; the error between the measured value and the theoretical value was less than ±1%, confirming the RSM approach was appropriate for optimizing plastein reaction of the ACE inhibitory peptides from chicken serum protein. Similarly, a study also used RSM to optimize the reaction conditions of plastein reaction for modification ACE inhibitory peptides from casein hydrolysate, which could enhance ACE inhibitory activity by 20.3% (Cao et al., 2018).

## 6 CONCLUSION

Chicken plasma protein was hydrolyzed by trypsin to obtain CPPH with ACE inhibitory activity of 53.5% ± 0.14%, and the hydrolysates were modified by a trypsin‐catalyzed plastein reaction to improve its ACE inhibitory activity. To improve the ACE inhibitory activity of CPPH, the conditions of trypsin‐catalyzed plastein reaction were optimized by single‐factor experiments and response surface methodology, and the resulting optimal conditions were as following: reaction time 4.91 hr, pH 7.98, and E/S ratio 5,682.12 U·g^−1^, temperature 40°C. Under the optimized conditions, ACE inhibitory rate reached 82.07% ± 0.03%, with an increase by 28.57%. The result showed that the trypsin‐catalyzed plastein reaction for CPPH could enhance ACE inhibitory activity of modified hydrolysates, indicating that chicken plasma protein has the potential in the development of new functional foods.

## CONFLICTS OF INTERESTS

The authors declare that there is no conflict of interest.

## AUTHORS’ CONTRIBUTIONS

The initiative for this work was from Zhongren Ma. Zhongren Ma and Dandan Gao designed experiments. Dandan Gao and Penghui Guo did CPPH modification. Xin Cao, Lili Ge, Hongxin Ma, Hao Cheng, and Yiqiang Ke prepared CPP, Shien Chen, Gongtao Ding, Ruofei Feng, and Zilin Qiao, prepared CPPH, Jialin Bai, and Nurul Izza Nordin did the determination of ACE inhibitory activity. Dandan Gao wrote the manuscript. All authors read and approved the final manuscript.

## ETHICAL REVIEW

This study does not involve any human or animal testing.
